# Immune Characteristics of Patients with Coronavirus Disease 2019 (COVID-19)

**DOI:** 10.14336/AD.2020.0317

**Published:** 2020-03-17

**Authors:** Xiaotian Dong, Mengyan Wang, Shuangchun Liu, Jiaqi Zhu, Yanping Xu, Hongcui Cao, Lanjuan Li

**Affiliations:** ^1^Department of Laboratory Medicine, The First Affiliated Hospital, College of Medicine, Zhejiang University, Hangzhou, China.; ^2^Department of Infectious Diseases, Xixi Hospital of Hangzhou, Hangzhou, China.; ^3^State Key Laboratory for Diagnosis and Treatment of Infectious Diseases, The First Affiliated Hospital, College of Medicine, Zhejiang University, Hangzhou, China.; ^4^National Clinical Research Center for Infectious Diseases, Hangzhou, China.; ^5^Zhejiang Provincial Key Laboratory for Diagnosis and Treatment of Aging and Physic-chemical Injury Diseases, Hangzhou, China

**Keywords:** coronavirus disease 2019 (COVID-19), immune characteristics, lymphocyte subsets, serum potassium

## Abstract

Up to now, little is known about the detailed immune profiles of COVID-19 patients from admission to discharge. In this study we retrospectively reviewed the clinical and laboratory characteristics of 18 COVID-19 patients from January 30, 2020 to February 21, 2020. These patients were divided into two groups; group 1 had a severe acute respiratory syndrome coronavirus 2 nucleic acid-positive duration for more than 15 days (n = 6) and group 2 had a nucleic acid-positive duration for less than 15 days (n = 12). Group 1 patients had lower level of peripheral blood lymphocytes (0.40 vs. 0.78 ×10^9^/L, *p = 0.024*) and serum potassium (3.36 vs. 3.79 mmol/L,* p = 0.043*) on admission but longer hospitalization days (23.17 vs. 15.75 days, *p < 0.001*) compared to Group 2 patients. Moreover, baseline level of lymphocytes (r = -0.62, *p* = 0.006) was negatively correlated with the nucleic acid-positive duration. Additionally, lymphocytes (420.83 vs. 1100.56 /μL), T cells (232.50 vs. 706.78 /μL), CD4^+^ T cells (114.67 vs. 410.44 /μL), and CD8^+^ T cells (94.83 vs. 257.44 /μL) in the peripheral blood analyzed by flow cytometry were significantly different between Group 1and Group 2. Furthermore, there was also a negative correlation between lymphocytes (r = -0.54, *p* = 0.038) or T cells (r = -0.55, *p* = 0.034) at diagnosis and the nucleic acid-positive duration, separately. In conclusion, the patients with nucleic acid-positive ≥ 15 days had significantly decreased lymphocytes, T cell and its subsets compared to those who remained positive for less than 15 days.

In late December 2019, several cases of unexplained pneumonia were found in some hospitals in Wuhan, Hubei Province, China [1]. Gene sequencing revealed a novel coronavirus, which led to severe pneumonia. Subsequently, the virus was named as severe acute respiratory syndrome corona virus 2 (SARS-CoV-2, previously known as 2019 novel coronavirus and 2019-nCoV) by the coronavirus study group (CSG) of the international committee on taxonomy of viruses [2], and the pneumonia disease caused by this virus was collectively called coronavirus disease 2019 (COVID-19) by the World Health Organization (WHO). COVID-19 has now caused an outbreak in China and other countries (www.who.int/emergencies/diseases/novel-coronavirus-2019) because of the lack of vaccines and effective antiviral drugs.

**Table 1:** Characteristics of COVID-19 patients on admission.

Characteristics	COVID-19 patients (n = 18)	Nucleic acid-positive <15 days (n = 12)	Nucleic acid-positive≥15 days (n = 6)	*p* value^1^
Age (years)	58.39 ± 17.21	56.50 ± 16.54	62.17 ± 19.47	0.527
Male, no. (%)	11 (61.11)	7 (58.33)	4 (66.67)	1.000
Hospitalization days	18.22 ± 4.86	15.75 ± 3.55	23.17 ± 2.93	<0.001*
SARS-CoV-2 nucleic acid-positive duration (day)	11.89 ± 7.28	7.50 ± 3.40	20.67 ± 4.03	<0.001*
Laboratory data				
Activated partial thromboplastin time (sec)	31.55 ± 6.04	30.68 ± 3.34	33.30 ± 9.70	0.401
International normalized ratio	0.99 ± 0.10	0.97 ± 0.06	1.05 ± 0.15	0.130
Prothrombin time (sec)	11.92 ± 1.20	11.62 ± 0.72	12.53 ± 1.76	0.130
D-dimer (μg/L)	877.56 ± 983.70	948.25 ± 1090.61	798.34 ± 325.92	0.680
White blood cell (10^9^/L)	9.17 ± 5.24	9.29 ± 5.21	8.93 ± 5.80	0.896
Neutrophils (10^9^/L)	8.16 ± 5.14	8.08 ± 5.21	8.30 ± 5.48	0.936
Lymphocytes (10^9^/L)	0.66 ± 0.46	0.78 ± 0.49	0.40 ± 0.27	0.024*
Hemoglobin (g/L)	131.17 ± 16.38	134.33 ± 15.95	124.83 ± 16.73	0.258
Monocytes (10^9^/L)	0.33 ± 0.23	0.39 ± 0.25	0.22 ± 0.10	0.144
Procalcitonin (ng/mL)	0.09 ± 0.09	0.09 ± 0.09	0.10 ± 0.09	0.899
Lactate dehydrogenase (U/L)	317.78 ± 136.18	305.42 ± 76.46	342.50 ± 221.57	0.601
Total bilirubin (μmol/L)	13.16 ± 7.56	11.94 ± 7.52	15.58 ± 7.70	0.351
Alanine aminotransferase (U/L)	25.22 ± 13.52	27.00 ± 13.71	21.67 ± 13.60	0.447
Albumin (g/L)	37.61 ± 5.00	38.55 ± 5.66	35.72 ± 2.82	0.269
Glutamyl transpeptidase (U/L)	45.78 ± 32.43	43.58 ± 24.99	50.17 ± 46.55	0.697
Alkaline phosphatase (U/L)	76.00 ± 31.60	81.42 ± 34.48	65.17 ± 23.86	0.318
Creatinine (μmol/L)	86.83 ± 67.25	94.08 ± 81.24	72.33 ± 21.91	0.534
Aspartate aminotransferase (U/L)	26.56 ± 14.02	26.17 ± 10.05	27.33 ± 21.09	0.874
C reactive protein (mg/L)	36.70 ± 44.02	28.68 ± 38.61	52.74 ± 53.33	0.288
Blood lactic acid (mmol/L)	2.13 ± 0.94	2.14 ± 1.16	2.10 ± 0.30	0.933
Partial pressure of oxygen (mmHg)	96.55 ± 47.30	108.66 ± 50.37	72.33 ± 31.14	0.128
Oxygen saturation (%)	94.35 ± 7.07	96.51 ± 2.73	90.03 ± 10.95	0.210
Hematocrit (%)	41.76 ± 7.57	42.02 ± 6.90	41.25 ± 9.48	0.847
Potassium (mmol/L)	3.65 ± 0.44	3.79 ± 0.33	3.36 ± 0.52	0.043*
Sodium (mmol/L)	138.11 ± 10.44	139.75 ± 3.79	134.83 ± 17.87	0.533
Creatine kinase (U/L)	22.33 ± 7.68	22.25 ± 8.57	22.50 ± 6.22	0.950

Categorical data are showed as number (%). Continuous data are showed as mean ±standard deviation (SD). Fisher's exact test, Mann-Whitney U test and Student’s t test were used to compare the two groups. *p <0.05. ^1^p value between the two groups (nucleic acid-positive < 15 days vs. nucleic acid-positive ≥ 15 days).

Previously, there were two types of coronaviruses, namely as severe acute respiratory syndrome-coronavirus (SARS-CoV) and Middle East respiratory syndrome-coronavirus (MERS-CoV), and they caused a previous epidemic of SARS and MERS in the population and led to severe respiratory diseases associated with high morbidity and mortality [3-6]. The clinical symptoms of COVID-19 are partly different from SARS and MERS; it can rapidly progress to acute respiratory distress syndrome (ARDS), multiple organ failure, or even death [7,8]. Huang et al had depicted the profile of the plasma cytokines of some severe COVID-19 patients who required intensive care unit (ICU) admission and revealed the existence of cytokine storm that was also observed in SARS and MERS patients [9]. Importantly, the cytokine storm caused by dysregulated immune response is the culprit of coronaviruses-related ARDS and multiple organ failure [10]. Thus, it is necessary to study the characteristics of immune cells in patients with COVID-19.

However, flow cytometry analysis is not a routine test for COVID-19 patients during clinical treatment. Therefore, little is known about the detailed immune profiles of COVID-19 patients from admission to discharge. In order to study the immune characteristics of COVID-19 patients, we retrospectively collected the data of clinical examination, laboratory characteristics, and flow cytometry data of peripheral blood lymphocytes of confirmed cases in the First Affiliated Hospital, College of Medicine, Zhejiang University.

## MATERIALS AND METHODS

### Patients

We retrospectively reviewed the clinical data of all COVID-19 patients admitted to the First Affiliated Hospital, Zhejiang University from January 30, 2020 to February 21, 2020. Finally, 18 COVID-19 patients who had complete data of peripheral blood lymphocyte subsets were enrolled. There were no AIDS patients in these 18 patients. The examination data of these patients were recorded from admission to discharge (the last discharge date was March 5). COVID-19 patients were diagnosed based on epidemiological history, clinical manifestations, and SARS-CoV-2 nucleic acid positive test of sputum and/or throat swabs and/or nasal swabs and/or stool samples through real-time RT-PCR, according to WHO Interim Guidance.[11] The present study was performed in accordance with the Helsinki Declaration and was approved by the Ethics Committee on Clinical Research of the First Affiliated Hospital, Zhejiang University (China). Informed consent was waived due to its retrospective nature.

### Data collection

We retrospectively collected general clinical records (age, sex, clinical diagnosis) of these patients and laboratory data during hospitalization. The flow cytometry assay data were gathered on admission and at hospital discharge. Other laboratory data were gathered at admission. Survival data were not collected because no patients (including critically ill) died after doctor's effective treatment.

### Study design

The 18 patients were divided into two groups, group 1 had a SARS-CoV-2 nucleic acid-positive duration of 15 days or more (nucleic acid-positive ≥ 15 days) and group 2 had a SARS-CoV-2 nucleic acid-positive duration of less than 15 days (nucleic acid-positive < 15 days). Routine laboratory data on admission and the first and last flow cytometry assay data from diagnosis to discharge of two groups were compared.

### Statistical analysis

Continuous variables with normal distribution were compared by Student’s t test or paired t-test. Otherwise, Mann-Whitney U test was used. And the continuous variables were expressed as mean ± standard deviation (SD). Categorical variables were compared by Fisher's exact test and expressed as frequencies and percentages. Spearman correlation analysis was used to determine the association between SARS-CoV-2 nucleic acid-positive duration and several items. All statistical analyses were performed using SPSS (version 21.0; IBM Corp., Armonk, NY, USA).

**Figure 1.**
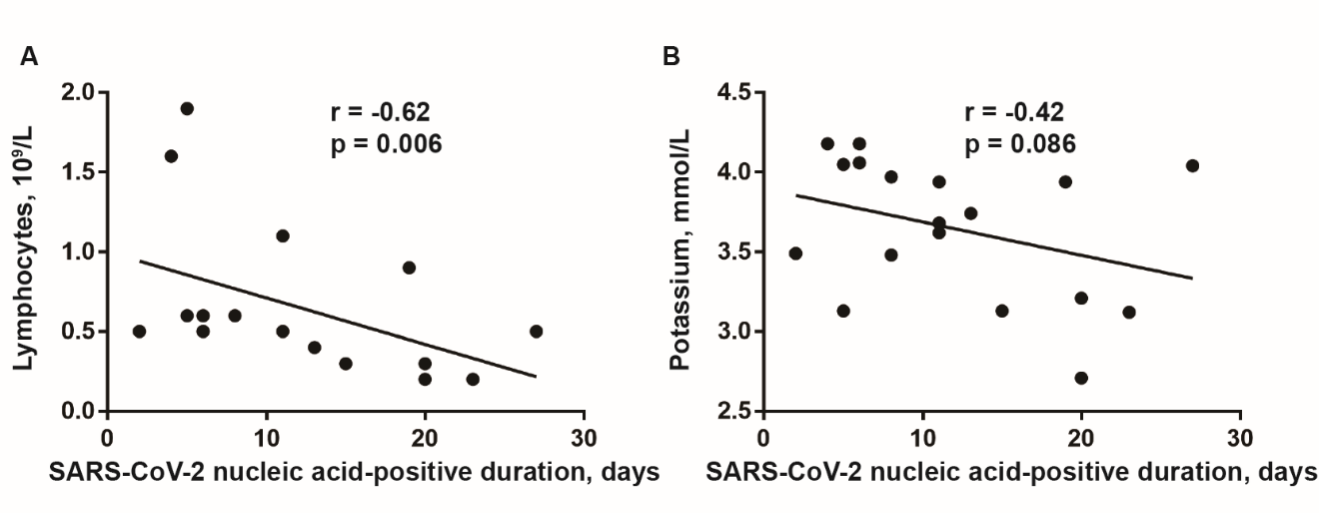
Correlation analysis between lymphocytes count and the duration of nucleic acid-positive of COVID-19 patients. Lymphocytes count in peripheral blood (A) but not serum potassium level (B) at admission was negatively correlated with the SARS-CoV-2 nucleic acid-positive duration. Spearman correlation analysis was performed. *p<0.05* as statistical significance.

**Table 2:** Characteristics of serum cytokines of COVID-19 patients at diagnosis.

Cytokines	Normal range	COVID-19 patients (n = 17)	Nucleic acid-positive < 15 days (n = 11)	Nucleic acid-positive ≥ 15 days (n = 6)	*p* value^1^
IFN-γ (pg/mL)	0.00-20.06	13.08 ± 15.96	9.06 ± 8.69	20.45 ± 23.75	0.302
IL-10 (pg/mL)	0.00-2.31	5.83 ± 4.91	4.15 ± 3.29	8.92 ± 6.14	0.052
IL-2 (pg/mL)	0.00-4.13	1.26 ± 0.55	1.08 ± 0.36	1.6 ± 0.71	0.143
IL-4 (pg/mL)	0.00-8.37	2.05 ± 1.93	1.50 ± 0.24	3.05 ± 3.14	0.281
IL-6 (pg/mL)	0.00-6.61	29.51 ± 19.10	23.82 ± 15.38	39.94 ± 22.20	0.097
TNF-α (pg/mL)	0.00-33.27	38.44 ± 41.46	22.49 ± 20.92	67.69 ± 55.14	0.103

Abbreviations: IFN-γ, interferon-γ; IL-6, interleukin-6; TNF-α, tumor necrosis factor-α Normal continuous data are showed as mean ±standard deviation (SD). Student’s t test was used to compare the two groups. *p <0.05 ^1^p value between the two groups (nucleic acid-positive < 15 days vs nucleic acid-positive ≥ 15 days).

## RESULTS

### Baseline levels of lymphocytes and serum potassium were significantly different within two groups of COVID-19 patients 

18 patients with COVID-19 were completely evaluated on admission including blood cells count, coagulation function test, blood chemistry (liver, lung, heart, and kidney function) and inflammation related indexes tests. Among them, 6 patients remained nucleic acid-positive for 15 days or more (Group 1, n = 6) and 12 patients remained positive for less than 15 days (Group 2, n = 12). Though most of the baseline indexes were insignificant between the two groups, lymphocytes (0.40 vs. 0.78 ×10^9^/L, *p* = 0.024), serum potassium (3.36 vs. 3.79 mmol/L, *p* = 0.043), and hospitalization days (23.17 vs. 15.75 days, *p* < 0.001) were significantly different between Group 1 and Group 2 (Table 1).

### Baseline level of lymphocytes was negatively correlated with the nucleic acid-positive duration 

To explore the potential correlation of nucleic acid-positive duration with other indexes, baseline levels of lymphocytes and serum potassium were both complied with the nucleic acid-positive days using spearman correlation analysis, respectively. Finally, lymphocytes (r = -0.62, *p* = 0.006) but not serum potassium level (r = -0.42, *p* = 0.086) on admission were negatively and significantly correlated with the nucleic acid-positive duration (Fig. 1).

**Table 3:** Lymphocyte subsets in COVID-19 patients at diagnosis and at discharge.

Lymphocyte subsets	Normal range	COVID-19 patients (n = 15)	Nucleic acid-positive < 15 days (n = 9)	Nucleic acid-positive ≥ 15 days (n = 6)	*p* value^†^
		At diagnosis	At discharge^1^	At diagnosis	At discharge^2^	At diagnosis	At discharge^3^	
Lymphocytes (/μL)	1530-3700	828.67 ± 700.99	1301.53 ± 566.77*	1100.56 ± 784.76	1372.89 ± 558.81	420.83 ± 240.61	1194.50 ± 613.92*	0.035*
T cells (/μL)	955-2860	517.07 ± 496.82	906.87 ± 412.71*	706.78 ± 567.01	949.44 ± 424.35	232.50 ± 121.53	843.00 ± 425.02*	0.038*
CD3^+^CD4^+^CD8^-^ T cells (/μL)	550-1440	292.13 ± 323.05	524.07 ± 294.16*	410.44 ± 375.48	548.67 ± 332.58	114.67 ± 60.32	487.17 ± 250.17*	0.047*
CD3^+^CD4^-^CD8^+^ T cells (/μL)	320-1250	192.40 ± 170.89	324.53 ± 186.26*	257.44 ± 191.84	339.33 ± 147.95	94.83 ± 62.08	302.33 ± 247.24	0.039*
CD4^+^/CD8^+^ ratio	0.71-2.78	1.69 ± 1.11	2.05 ± 1.18	1.81 ± 1.27	1.86 ± 1.13	1.51 ± 0.90	2.32 ± 1.30	0.629
B cells (/μL)	90-560	168.40 ± 135.75	221.73 ± 111.09*	219.44 ± 148.39	226.44 ± 131.06	91.83 ± 68.10	214.67 ± 83.52*	0.072
NK cells (/μL)	150-1100	120.13 ± 82.92	140.93 ± 123.40	148.56 ± 91.59	179.78 ± 118.71	77.50 ± 46.82	82.67 ± 115.31	0.106

Normal continuous data are showed as mean ±standard deviation (SD). Paired t test and Student’s t test were used to compare the two groups. *p<0.05. 1p value between COVID-19 patients at diagnosis and at discharge, Paired t test. 2p value between nucleic acid-positive < 15 days patients at diagnosis and at discharge, Paired t test. 3p value between nucleic acid-positive ≥ 15 days patients at diagnosis and at discharge, Paired t test. †p value between the two groups (nucleic acid-positive < 15 days vs. nucleic acid-positive ≥ 15 days) at diagnosis. Student’s t test.

### T cells but not B cells or natural killer cells at diagnosis negatively correlated with the nucleic acid-positive duration

**Figure 2.**
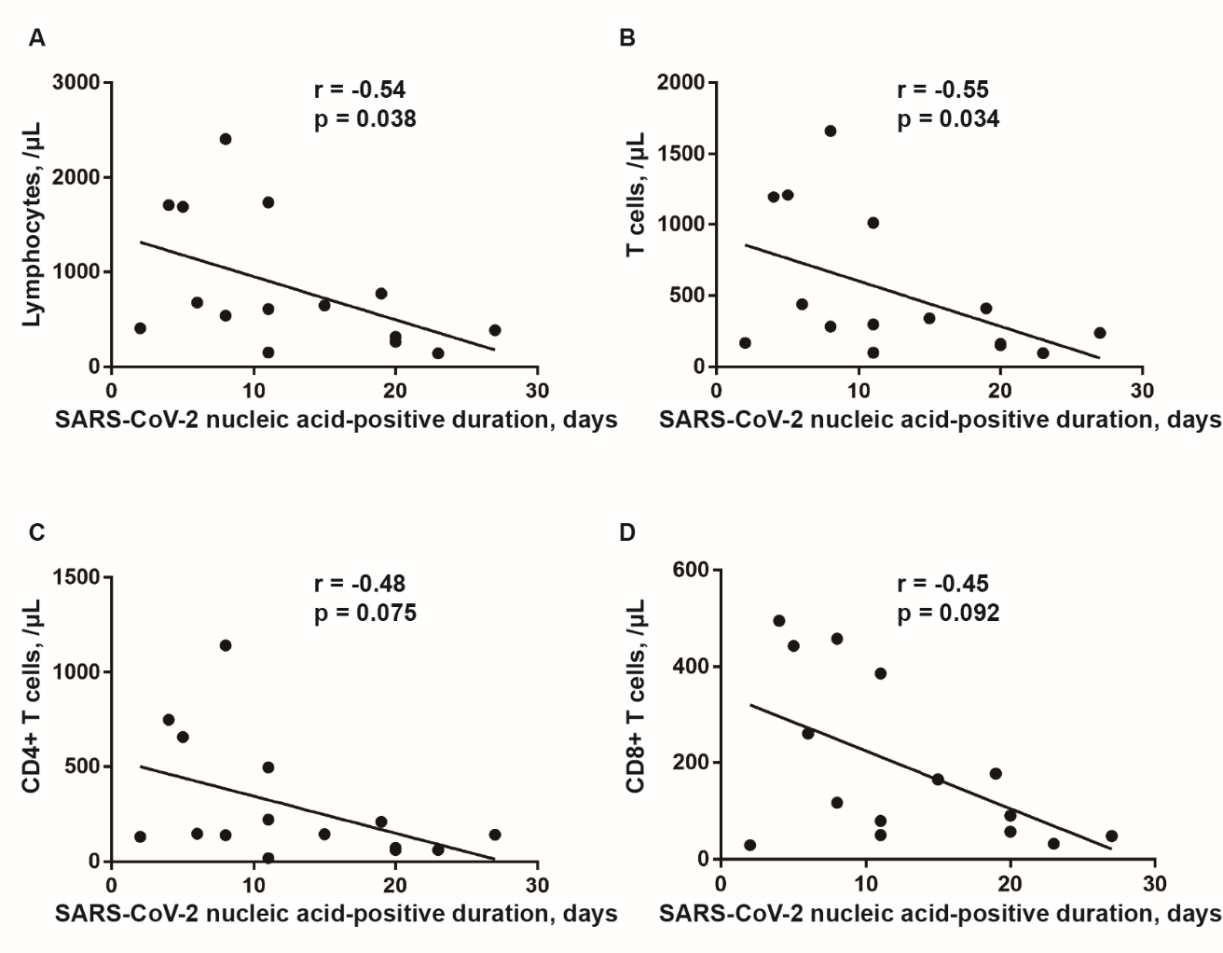
Correlation analysis between lymphocyte subset numbers and the nucleic acid-positive duration of COVID-19 patients. Lymphocytes count in peripheral blood (A) and T cells (B) at diagnosis were negatively correlated with the duration of SARS-CoV-2 nucleic acid-positive. CD4^+^ T cells (C) and CD8^+^ T cells (D) were not correlated with the duration of SARS-CoV-2 nucleic acid-positive. Spearman correlation analysis was performed. *p<0.05* as statistical significance.

Importantly, peripheral blood lymphocyte subsets and serum cytokines of the two groups were evaluated. There was no significant difference of cytokines at diagnosis between two groups (Table 2). However, the average levels of interleukin-6 (IL-6, 29.51 pg/mL), IL-10 (5.83 pg/mL) and tumor necrosis factor-α (TNF-α, 38.44 pg/mL) of the 17 patients (one patient was not subjected to cytokines analysis) were elevated and compared to the normal population. Otherwise, lymphocytes (420.83 vs. 1100.56/μL), T cells (232.50 vs. 706.78/μL), CD4^+^ T cells (114.67 vs. 410.44/μL) and CD8^+^ T cells (94.83 vs. 257.44 /μL) were significantly different between group 1 and group 2 at diagnosis (Table 3). Moreover, there was no significant difference with respect to immune cell counts from diagnosis to discharge for group 2 patients. But group 1 patients (nucleic acid-positive ≥ 15 days) showed significant elevations of lymphocytes (420.83 vs. 1194.50/μL), T cells (232.50 vs. 843.00 /μL), CD4^+^ T cells (114.67 vs. 487.17/μL), and B cells (91.83 vs. 214.67/μL) at diagnosis compared to those at discharge (Table 3). Similarly, spearman correlation analysis was also performed and revealed that only lymphocytes (r = -0.54, *p* = 0.038) and T cells (r = -0.55, *p* = 0.034) at diagnosis were negatively correlated with the nucleic acid-positive duration (Fig. 2).

### Discussion

In this study, we retrospectively reviewed and analyzed the characteristics of peripheral blood lymphocyte subsets and serum cytokines of 18 patients with COVID-19 who were hospitalized in the First Affiliated Hospital, Zhejiang University. We reviewed the alterations of immune profiles of COVID-19 patients from admission to discharge. And we also explored the relationship between the duration of SARS-CoV-2 nucleic acid-positive and immune cells subsets.

Since COVID-19 epidemics occurred in China, many types of investigations have greatly enriched our knowledge about its epidemiological and clinical characteristics as well as genomic characterization [7-9,12]. Then, the Chinese government has launched a series of researches and development support programs for COVID-19 vaccine, drugs and testing reagents.

However, we still didn’t have effectively preventive or therapeutic agents for anti-SARS-CoV after a decade of SARS outbreak [13]. Fortunately, there are many similarities between SARS and COVID-19 patients (for example: fever, dry cough, pulmonary infiltrates, lymphopenia, elevated cytokines, immune cells infiltration) [7,9,14-16]. Probably because SARS-CoV and SARS-CoV-2 are both coronaviruses, they were able to bind the angiotensin I converting enzyme 2 (ACE2) receptor in the target cells during invasion despite their different genome sequences [12,17,18]. Thus, it is important to further study SARS and SARS-CoV. In SARS patients, researchers had discovered reduction of lymphocytes, T cell and its subsets, B cells and natural killer (NK) cells in acute phase and restoration of these immune cells after recovery [19,20]. Severe lung injury of SARS patients was believed to result from immune dysregulation which was commonly seen in prolonged virus clearance [21]. Importantly, the decrease of T cell and its subsets were associated with the severity of SARS patients [20,22]. A recent study displayed that transplantation of ACE2^-^ mesenchymal stem cells could improve the outcome of COVID-19 patients by immunoregulation [23]. These results suggested the importance of immune cells in antivirual process. In our study, we also observed decreased B cells (5/15 of patients), NK cells (10/15 of patients), T cells (11/15 of patients), CD4^+^ T cells (12/15 of patients), CD8^+^ T cells (11/15 of patients), and lymphocytes (11/15 of patients). And the number of T cells but not B cells or NK cells at diagnosis was negatively correlated with the nucleic acid-positive duration. However, further study about mortality and severity comparison between the two groups (nucleic acid-positive < 15 days and ≥ 15 days) was needed.

There are several limitations in this study. First, although we retrospectively reviewed the clinical data of all COVID-19 patients from January 30, 2020 to February 21, 2020, only 18 patients who had flow cytometry detection data were enrolled, and 1 patient did not execute cytokines analysis and 3 patients did not execute immune cells analysis. Thus, we suggest that the flow cytometry detection should be complied as a routine test for COVID-19 patients during clinical treatment, and further in-depth research is need. Secondly, there was no data of SARS-CoV-2 nucleic acid load because a qualitative test but not a quantitative test on nucleic acid was used in the clinical diagnosis. And nucleic acid load may be different between the two groups on admission.

In general, the patients with nucleic acid-positive ≥ 15 days had significantly decreased lymphocytes, T cell and its subsets compared with group nucleic acid-positive <15 days. The level of lymphocytes and T cells at diagnosis was negatively correlated with the nucleic acid-positive duration of COVID-19 patients, separately.
